# The effects of elevated CO_2_ concentration on competitive interaction between aceticlastic and syntrophic methanogenesis in a model microbial consortium

**DOI:** 10.3389/fmicb.2014.00575

**Published:** 2014-10-30

**Authors:** Souichiro Kato, Rina Yoshida, Takashi Yamaguchi, Tomoyuki Sato, Isao Yumoto, Yoichi Kamagata

**Affiliations:** ^1^Bioproduction Research Institute, National Institute of Advanced Industrial Science and TechnologySapporo, Japan; ^2^Division of Applied Bioscience, Graduate School of Agriculture, Hokkaido UniversitySapporo, Japan; ^3^Research Center for Advanced Science and Technology, The University of TokyoTokyo, Japan; ^4^Department of Civil and Environmental Engineering, Nagaoka University of TechnologyNagaoka, Japan

**Keywords:** model consortia, methanogenesis, acetate, thermodynamics, CO_2_ concentration

## Abstract

Investigation of microbial interspecies interactions is essential for elucidating the function and stability of microbial ecosystems. However, community-based analyses including molecular-fingerprinting methods have limitations for precise understanding of interspecies interactions. Construction of model microbial consortia consisting of defined mixed cultures of isolated microorganisms is an excellent method for research on interspecies interactions. In this study, a model microbial consortium consisting of microorganisms that convert acetate into methane directly (*Methanosaeta thermophila*) and syntrophically (*Thermacetogenium phaeum* and *Methanothermobacter thermautotrophicus*) was constructed and the effects of elevated CO_2_ concentrations on intermicrobial competition were investigated. Analyses on the community dynamics by quantitative RT-PCR and fluorescent *in situ* hybridization targeting their 16S rRNAs revealed that high concentrations of CO_2_ have suppressive effects on the syntrophic microorganisms, but not on the aceticlastic methanogen. The pathways were further characterized by determining the Gibbs free energy changes (ΔG) of the metabolic reactions conducted by each microorganism under different CO_2_ concentrations. The ΔG value of the acetate oxidation reaction (*T. phaeum*) under high CO_2_ conditions became significantly higher than -20 kJ per mol of acetate, which is the borderline level for sustaining microbial growth. These results suggest that high concentrations of CO_2_ undermine energy acquisition of *T. phaeum*, resulting in dominance of the aceticlastic methanogen. This study demonstrates that investigation on model microbial consortia is useful for untangling microbial interspecies interactions, including competition among microorganisms occupying the same trophic niche in complex microbial ecosystems.

## INTRODUCTION

In natural and engineered environments, many species of microorganisms coexist by interacting with each other. Comprehension of interspecies interactions is essential for describing the features of complex microbial ecosystems, and competition among microorganisms occupying similar trophic niches is a conventional and significant aspect of such interspecies interaction. Coexistence of multiple microorganisms with similar trophic niches is regarded as one of the major factors to confer functional stability and resiliency on microbial ecosystems (Loreau et al., 2001; Deng, 2012). It is important to grasp how the population of each microorganism changes depending on a specific environmental disturbance. Most microbial ecological research has assessed the effects of specific environmental factors on competitive interactions among multiple microbial species by observing the transition of abundances of each microorganism responding to environmental disturbances. Although this approach has produced many excellent outcomes, existence of non-target microorganisms and uncontrollable environmental factors in the systems often hamper precise understanding of the effects of specific environmental factors on the competitive interactions among target microorganisms.

Construction of microbial model consortia, in which interspecies interactions in ecosystems are reproduced by defined co-culture of isolated microorganisms, is appreciated as a worthwhile method to investigate microbial interactions (Haruta et al., 2009; De Roy et al., 2014; Großkopf and Soyer, 2014). For instance, the complex phenomenon of bacterial competition as being similar to rock-paper-scissors among colisin-producing, colisin-resistant, and colisin-sensitive strains was untangled by constructing model co-culture systems (Kerr et al., 2002; Nahum et al., 2011). Kato et al. (2005, 2008) constructed model microbial consortia composed of 4–5 bacterial strains, in which all members stably coexisted for long period of time, and demonstrated that existence of both positive and negative interspecies interactions among the members make these consortia stable. The construction of model consortia is a specific and beneficial feature of microbiological research fields, which will also be effective for proof-of-concept studies for theories in the field of macro-ecology (Haruta et al., 2009, 2013).

Methanogenesis from organic compounds is a complex microbial process accomplished by catabolic interactions among different trophic levels of microorganisms (Schink, 1997; Jones et al., 2008; Kato and Watanabe, 2010). Among the sequential biodegradation processes, acetate is the most important intermediary metabolite (Schink, 1997). Methanogenic acetate degradation proceeds by either aceticlastic methanogenesis or syntrophic acetate oxidation. The aceticlastic pathway is solely mediated by aceticlastic methanogens (Jetten et al., 1992). On the contrary, syntrophic acetate oxidation pathway requires cooperative interactions of two different types of microorganisms: acetate is first oxidized to H_2_ and CO_2_ by syntrophic acetate-oxidizing bacteria (SAOB), and then hydrogenotrophic methanogens convert the products to CH_4_ (Zinder and Koch, 1984). As the acetate oxidation reaction is endoergonic under the standard conditions and is feasible only under extremely low H_2_ partial pressure, acetate oxidation by SAOB requires H_2_ elimination by hydrogenotrophic methanogens (Karakashev et al., 2006; Hattori, 2008). These two different acetate-degrading methane-producing pathways and organisms involved can co-exist, but diverse environmental factors, such as temperature, pH, salinity, toxic compounds, and concentrations of substrates determine one pathway and organisms to dominate over the other (Nüsslein et al., 2001; Shigematsu et al., 2004; Karakashev et al., 2006; Hao et al., 2013; Kato et al., 2014).

In our previous studies, we demonstrated that the syntrophic pathway is the dominant methanogenic acetate degradation pathway in underground, thermophilic petroleum reservoirs (Mayumi et al., 2011). We further demonstrated that aceticlastic pathway becomes dominant under high CO_2_ concentrations, which mimicked carbon capture and storage field conditions (Mayumi et al., 2013), whereas syntrophic acetate oxidation dominated over aceticlastic reactions under low CO_2_ concentrations. Since CO_2_ is either substrate or product of aceticlastic methanogenesis, acetate oxidation, and hydrogenotrophic methanogenesis, high CO_2_ concentration alters the thermodynamics of each methanogenic reaction, which may cause the observed transition between syntrophic and aceticlastic methanogenic pathways. However, all the data were based on the analyses of complex microbial communities in field samples thus many other factors that affect the community shift could not be ruled out.

In the present study, the effect of CO_2_ concentrations on methanogenic microorganisms were assessed by using a defined inorganic medium and a defined methanogenic consortium which is comprised of three organisms, i.e., SAOB, hydrogenotrophic methanogen and aceticlastic methanogen, namely, which contains two different acetate-degrading methanogenic pathways. The experiments allowed to precisely show the CO_2_ concentrations to be a crucial factor affecting the dominance of respective pathways and organisms.

## MATERIALS AND METHODS

### MICROORGANISMS AND CULTURE CONDITIONS

*Methanosaeta thermophila* DSM6194^T^ (Kamagata and Mikami, 1991) and *Thermacetogenium phaeum* DSM12270^T^ (Hattori et al., 2000) were obtained from the Deutsche Sammlung von Mikroorganismen und Zellkukturen GmbH (Braunschweig, Germany). *Methanothermobacter thermautotrophicus* strain TM was isolated from a thermophilic anaerobic methanogenic reactor in Japan (Hattori et al., 2000). Routine cultivations were conducted at 55∘C with 68-ml capacity serum vials containing 20 ml of a bicarbonate-buffered inorganic medium (pH 7.0; Kato et al., 2014) under an atmosphere of N_2_-CO_2_ [80/20 (v/v)] without shaking. Pyruvate (40 mM) or 200 kPa H_2_-CO_2_ [80/20 (v/v)] was supplemented as energy and carbon sources for the pure cultures of *T. phaeum* and *Methanothermobacter thermautotrophicus*, respectively. Sodium acetate (40 mM) was utilized as an energy and carbon source for the pure culture of *Methanosaeta thermophila*, the defined co-culture of *T. phaeum* and *Methanothermobacter thermautotrophicus*, and the tri-culture of the three strains. The tri-culture was constructed by simultaneously inoculating 1 and 2 ml of the early-stationary phases of pure culture of *Methanosaeta thermophila* and the defined co-culture of *T. phaeum* and *Methanothermobacter thermautotrophicus,* respectively, into the 20 ml of the medium. Although the long term stability of the tri-culture was not been tested, coexistence of the three microorganisms in the batch culture was confirmed.

### CULTURES WITH DIFFERENT CO_2_ CONCENTRATIONS

Three culture conditions were prepared to examine the effects of CO_2_ concentrations on the microorganisms. For each condition, the media were supplemented with different concentrations of sodium bicarbonate and the gas phases were replaced with N_2_/CO_2_ mixed gas with different volume ratios, as described in **Table 1**. The medium was bubbled with the respective deoxygenated gas with 100 ml min^-1^ for 5 min and immediately capped with a butyl rubber stopper and an aluminum cap. The medium pH was adjusted to 7.0 by adding 1N NaOH solution before the cultivation, and the fluctuation of pH value throughout the cultivation was less than 0.2. For pH measurement, 100 μl of the medium was sampled with syringes and the pH value was determined using a compact pH meter B-212 (Horiba). The concentration of CO_2_ in the aqueous phase [*c_aq_* (M)] was calculated according to Henry’s law (*c_aq_* = *kp*), where *k* is the Henry’s low constant (0.019 for CO_2_ at 55∘C) and *p* is the partial pressure of CO_2_ in the gas phase (atm). Then the bicarbonate concentrations were calculated based on the equilibrium formula (H_2_CO_3_ = H^+^ + $HCO_{3}^{-}$) with the equilibrium constant of 4.47 × 10^7^. The culture experiments were conducted in triplicate and the student’s *t*-test was used for the statistical analyses.

**Table 1:** Media with different initial [ΣCO_**2**_] used in this study.

[ΣCO_2_]_initial_ (mmol l^-1^)	NaHCO_3_ added (mM)	Partial pressure of the gas phase CO_2_ (atm)	Calculated [$HCO_{3}^{-}$]_initial_ (mM)
5.0	5	0	0.8
50.7	35	0.2	8.1
113.4	35	1	18.1

Growth of *Methanosaeta thermophila* and *Methanothermobacter thermautotrophicus* in pure and mixed cultures was determined by measuring methane production. Growth of *T. phaeum* pure culture was determined by measuring acetate production from pyruvate. The partial pressure of CH_4_ was determined using a gas chromatograph GC-2014 (Shimadzu) as described previously (Kato et al., 2014). The partial pressure of H_2_ was determined using a trace reduction gas analyzer TRA-1000 (ACE Inc.) according to the manufacturer’s instruction. The concentrations of organic acids were determined using a high performance liquid chromatography (D-2000 LaChrom Elite HPLC system, HITACHI) equipped with Aminex HPX-87H Ion Exclusion column (BIO-RAD) and L2400 UV detector (HITACHI).

### FLUORESCENT *IN SITU* HYBRIDIZATION (FISH)

Microbial cells of the tri-cultures in the early stationary phases were collected by centrifugation, fixed with 4% paraformaldehyde in phosphate buffered saline (PBS; 137 mM NaCl, 2.7 mM KCl, 8.1 mM Na_2_HPO_4_, 1.5 mM KH_2_PO_4_, pH 7.2) and left for 6 h at 4∘C. The samples were washed three times with PBS, immobilized on glass slides, and dehydrated by successive passages through 50, 70, 80, 90, and 100% ethanol (3 min each). The following oligonucleotide probes complementary to specific regions of 16S rRNA were utilized for hybridizations: (i) Alexa488-labeled EUB338, specific for the domain *Bacteria* (Amann et al., 1990) and (ii) TexRed-labeled ARCH917, specific for the domain *Archaea* (Loy et al., 2002), (iii) Alexa594-labeled MSMX860, specific for the order *Methanosarcinales* (Raskin et al., 1994), and (iv) Alexa488-labeled MB311, specific for the order *Methanobacteriales* (Crocetti et al., 2006). Hybridizations were performed at 46∘C for 3 h with hybridization buffer (0.9 M NaCl, 0.1 M Tris-HCl, pH 7.5) containing 5 ng μl^-1^ of each labeled probe. The specificity of each probe was confirmed by FISH observations using pure cultures of the three microorganisms used in this study even with the hybridization buffer not containing formamide. The washing step was done at 48∘C for 30 min with washing buffer (0.2 M NaCl, 0.1 M Tris-HCl, pH 7.5). The samples hybridized with the probes were observed with a fluorescent microscope Provis AX70 (Olympus).

### QUANTITATIVE RT-PCR (qRT-PCR)

Microbial cells were harvested from the mid-logarithmic phases by centrifugation at 10,000 X *g* and 4∘C. Total RNA was isolated using ISOGEN II reagent (Nippon Gene, Japan) combined with a bead-beating method, as described previously (Kato et al., 2014). Total RNA was purified using an RNeasy Mini kit (Qiagen) with DNase treatment (RNase-free DNase set, Qiagen) as described in the manufacturer’s instructions. The purified RNA was spectroscopically quantified using a NanoDrop ND-1000 spectrophotometer (NanoDrop Technologies). The PCR primers used for quantitative RT-PCR (qRT-PCR) were designed with Primer3 software (http://simgene.com/Primer3) and are listed in **Table 2**. Quantification of 16S rRNA copy numbers in the defined mixed culture were performed by one-step real-time RT-PCR using a Mx3000P QPCR System (Stratagene) and RNA-direct SYBR Green Realtime PCR Master Mix (Toyobo) as described previously (Kato et al., 2014). At least three biological replicates were subjected to qRT-PCR analysis, and at least two separate trials were conducted for each sample. Standard curves were generated with serially diluted PCR products (10^3^–10^8^ copies ml^-1^) amplified using the respective primer sets and were used to calculate the copy number of rRNA in the total RNA samples.

**Table 2:** Quantitative RT-PCR (qRT-PCR) primers designed and used in this study.

Primer name	Sequence (5′–3′)	Target
PT387f	GATAAGGGGACCTCGAGTGCT	*Methanosaeta thermophila*
PT573r	GGCCGGCTACAGACCCT	*Methanosaeta thermophila*
PB486f	ACGGGACGAAGGGAGTGACGG	*Thermacetogenium phaeum*
PB646r	CTCCTCCCCTCAAGTCATCCAGT	*Thermacetogenium phaeum*
TM1139f	TTACCAGCGGAACCCTTATGG	*Methanothermobacter thermautotrophicus*
TM1275r	ACCTGGTTTAGGGGATTACCTCC	*Methanothermobacter thermautotrophicus*

## RESULTS AND DISCUSSION

### EFFECTS OF CO_2_ CONCENTRATIONS ON THE MODEL METHANOGENIC CONSORTIUM

As the model consortium performing methanogenic acetate degradation, we utilized a defined mixed culture of an aceticlastic methanogen (*Methanosaeta thermophila*), a hydrogenotrophic methanogen (*Methanothermobacter thermautotrophicus*), and a SAOB (*T. phaeum*; **Table 3**). These microbial species were originally isolated from a thermophilic methanogenic digester (Kamagata and Mikami, 1991; Hattori et al., 2000) and are regarded as representative species for the methanogenic acetate degradation reactions that occur in various natural environments such as high-temperature petroleum reservoirs (Pham et al., 2009; Mayumi et al., 2011, 2013) and thermophilic methanogenic digesters (Sekiguchi et al., 1998; McHugh et al., 2003; Hori et al., 2011).

**Table 3:** The metabolic reactions and the respective standard Gibbs free energy changes (ΔG∘’) of the microorganisms utilized in this study.

Microbial species	Metabolic reactions	ΔG^∘′^ (kJ mol^-1^)^a^
*Methanosaeta thermophila*	CH_3_COO^-^ + H_2_O → CH_4_ + $HCO_{3}^{-}$	-31.0
*Thermacetogenium phaeum*	CH_3_COO^-^ + 4H_2_O → 2$HCO_{3}^{-}$ + 4H_2_ + H^+^	+104.6
*Methanothermobacter thermautotrophicus*	4H_2_ + $HCO_{3}^{-}$ + H^+^ → CH_4_ + 3H_2_O	-135.6

*^a^The ΔG^∘′^ values were calculated according to the reference (Thauer et al., 1977).*

**FIGURE 1:**
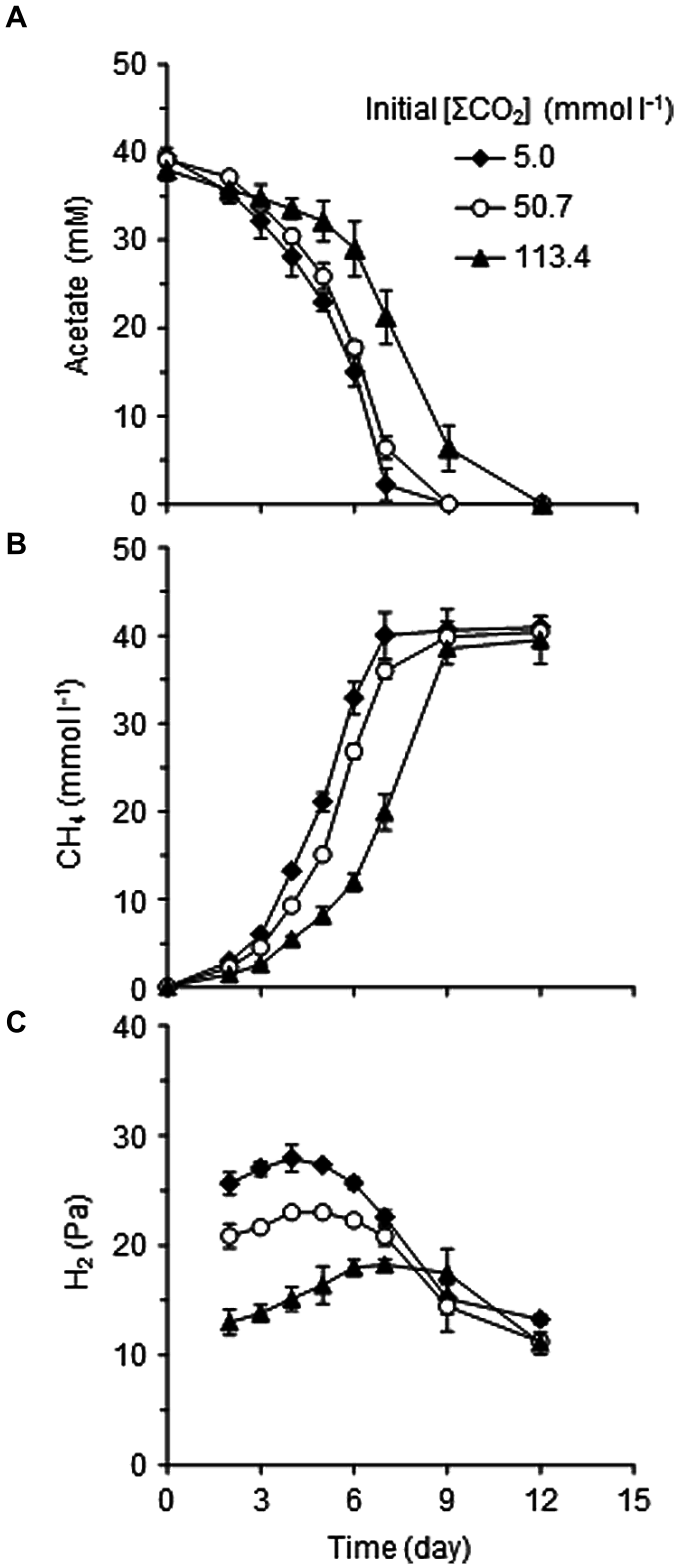
**Effects of CO_**2**_ concentrations on the metabolism of the model consortium composed of *Thermacetogenium phaeum*, *Methanothermobacter thermautotrophicus*, and *Methanosaeta thermophila*.** Time courses of acetate **(A)**, CH_4_ **(B),** and H_2_ **(C)** concentrations during cultivation on acetate with different [ΣCO_2_]_initial_ are shown. Data are presented as means of three independent cultures, and error bars represent SDs.

To adequately assess the effects of CO_2_ concentration itself, media with different supplementation of CO_2_/$HCO_{3}^{-}$ were prepared (**Table 1**). The initial concentrations of total CO_2_/$HCO_{3}^{-}$ in the cultures, designated as [ΣCO_2_]_initial_, were 5.0, 50.7, or 113.4 mmol l^-1^. The model consortium composed of *Methanosaeta thermophila*, *Methanothermobacter thermautotrophicus*, and *T. phaeum* was cultivated under the three different [ΣCO_2_]_initial_ conditions to evaluate their methanogenic acetate degradation abilities (**Figure 1**). A stoichiometric production of CH_4_ from acetate in a 1:1 molar ratio was observed in all culture conditions tested. Both acetate consumption and CH_4_ production rates slightly decreased with increasing the [ΣCO_2_]_initial_ (**Figures 1A,B**). Interestingly, the partial pressure of H_2_, which is an important intermediate of syntrophic acetate degradation, significantly decreased with increasing the [ΣCO_2_]_initial_ (**Figure 1C**). This observation suggests that syntrophic methanogenic microorganisms are influenced by elevated CO_2_ concentrations.

**FIGURE 2:**
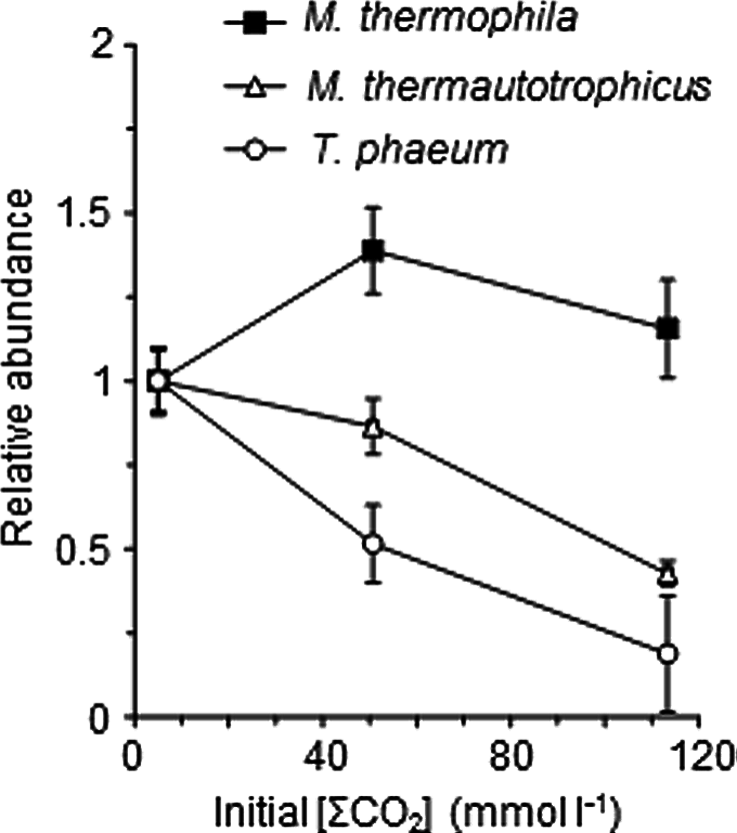
**Relative abundances of *T. phaeum*, *Methanothermobacter thermautotrophicus*, and *Methanosaeta thermophila* in the model consortium with different [ΣCO_**2**_]_**initial**_.** The 16S rRNA copy numbers of each microorganism in the mid-exponential phases were determined by the qRT-PCR analysis. The abundance of each microorganism was normalized against those of the cultures with [ΣCO_2_]_initial_ of 5.0 mmol l^-1^, and plotted against the respective [ΣCO_2_]_initial_ values. Data are presented as the means of three independent cultures, and error bars represent SDs.

**FIGURE 3:**
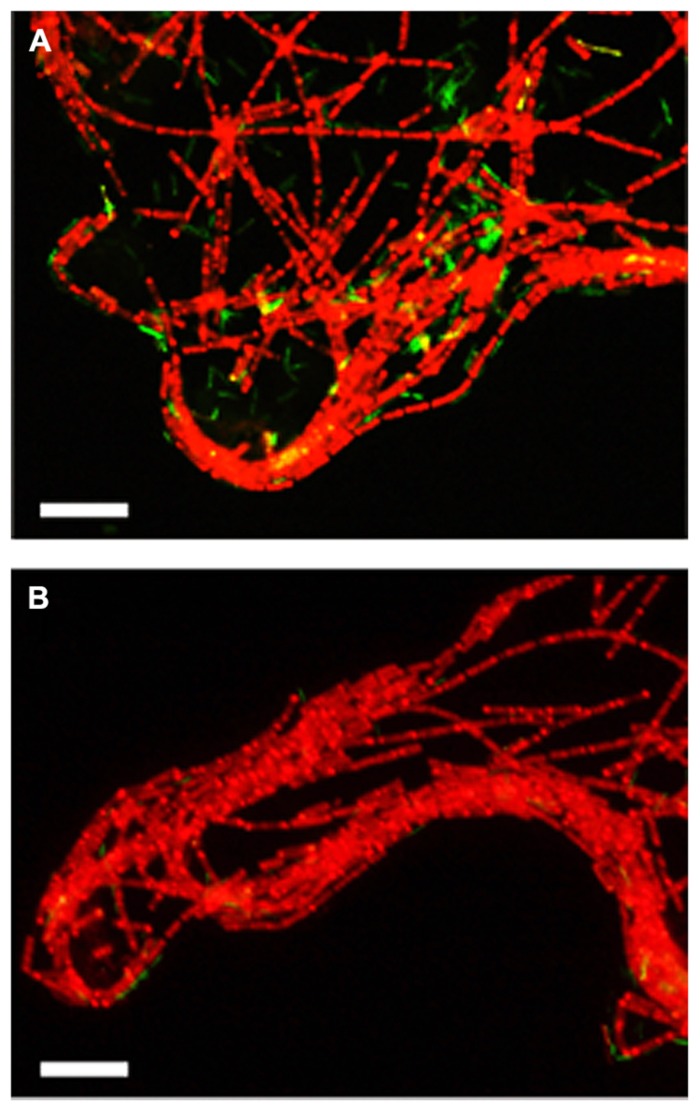
**Fluorescent microscopic images of the three-strains consortium cultivated with the [ΣCO_**2**_]_**initial**_ of 5.0 **(A)** or 113.4 mmol l^**–1**^**(B)**.** The exponential phase cultures were subjected to *in situ* hybridization with the probes described in the Materials and Methods section. Red, *Methanosaeta thermophila* (hybridized with Archaea917-TexRed and MSMX860-Alexa594); Yellow, *Methanothermobacter thermautotrophicus* (hybridized with Archaea917-TexRed and MB311- Alexa488); Green, *T. phaeum* (hybridized with EUB338-Alexa488). Bars = 10 μm.

To assess the influence of the elevated CO_2_ concentrations on each methanogenic pathway, the relative abundances of each microorganism in the exponentially growing cultures of the model consortium with the different [ΣCO_2_]_initial_ were evaluated by FISH and qRT-PCR analyses. The qRT-PCR analysis clearly demonstrated the decrease of the abundances of *Methanothermobacter thermautotrophicus* and *T. phaeum* in the higher [ΣCO_2_]_initial_ cultures (**Figure 2**). The FISH analysis also demonstrated that the relative abundances of *Methanothermobacter thermautotrophicus* and *T. phaeum* in the cultures with higher CO_2_ concentrations are significantly lower than those in the low CO_2_ cultures (**Figure 3**). These results indicate that the syntrophic methanogenic pathway is more strongly influenced by the elevation of CO_2_ concentrations compared to the aceticlastic pathway.

### EFFECTS OF CO_2_ CONCENTRATIONS ON THE ACETICLASTIC AND SYNTROPHIC PATHWAYS

**FIGURE 4:**
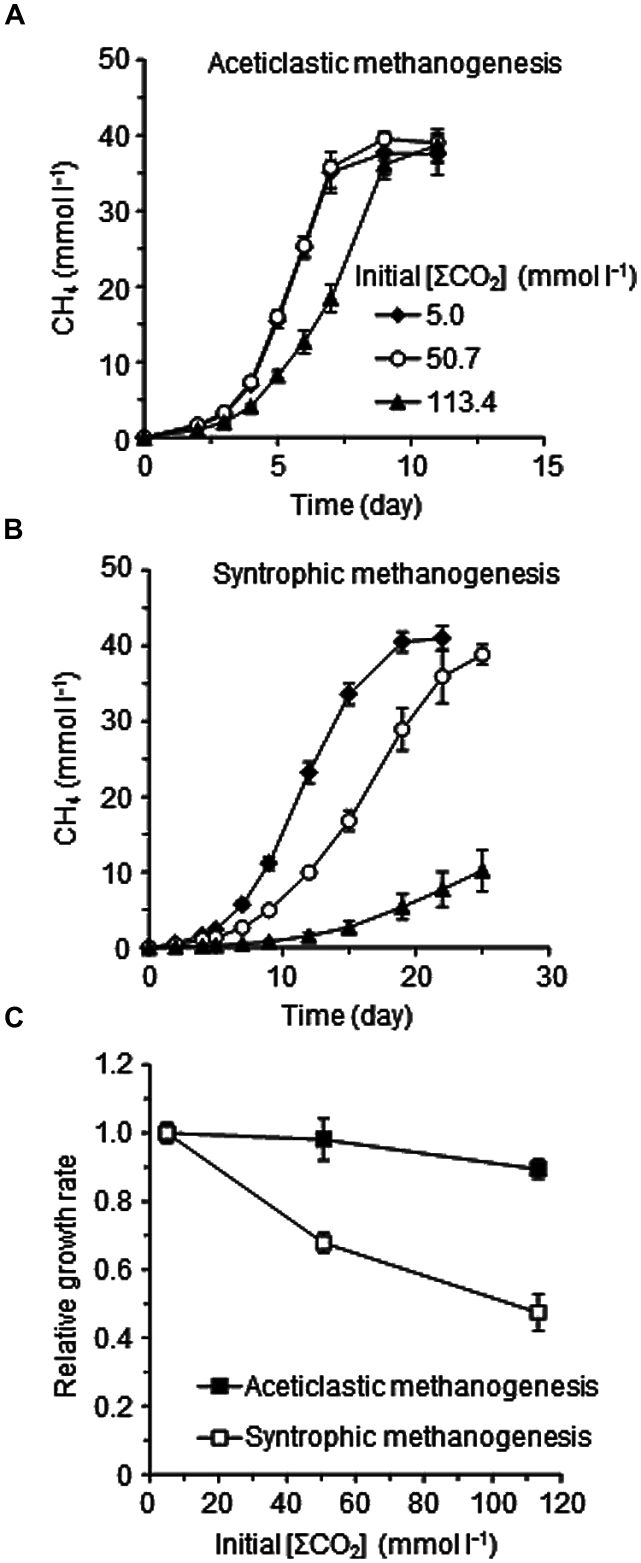
**Effects of CO_**2**_ concentrations on aceticlastic methanogenesis by the pure-culture of *Methanosaeta thermophila***(A)**, syntrophic methanogenesis by the defined co-culture of *T. phaeum* and *Methanothermobacter thermautotrophicus***(B)**, and the relative methanogenic rates of the aceticlastic and syntrophic methanogenic cultures **(C)**.** The methanogenic rates determined from respective methane generation data **(A,B)** were normalized against those of the cultures with [ΣCO_2_]_initial_ of 5.0 mmol l^-1^. Data are presented as the means of three independent cultures and error bars represent SDs.

To confirm the differences in the suppressive effects of elevated CO_2_ concentrations on the two methanogenic pathways, the pure culture of *Methanosaeta thermophila* and the defined co-culture of *Methanothermobacter thermautotrophicus* and *T. phaeum* were separately cultivated in the media with the different [ΣCO_2_]_initial_ (**Figure 4**). The growth of *Methanosaeta thermophila* was barely affected by the elevated CO_2_ concentration: the methanogenic rate in the [ΣCO_2_]_initial_ of 113.4 mmol l^-1^ cultures decreased only about 10% compared to the cultures with [ΣCO_2_]_initial_ of 5.0 mmol l^-1^ (**Figures 4A,C**). On the contrary, the methanogenic rate of the syntrophic co-culture in the [ΣCO_2_]_initial_ of 113.4 mmol l^-1^ dropped to less than half of that in the cultures with [ΣCO_2_]_initial_ of 5.0 mmol l^-1^ (**Figures 4B,C**). These observations confirm the assumption that the syntrophic acetate degradation pathway is more susceptible to elevated CO_2_ concentrations than the aceticlastic pathway.

### EFFECTS OF CO_2_ CONCENTRATIONS ON THE PURE CULTURES OF *Methanothermobacter thermautotrophicus* AND *T. phaeum*

**FIGURE 5:**
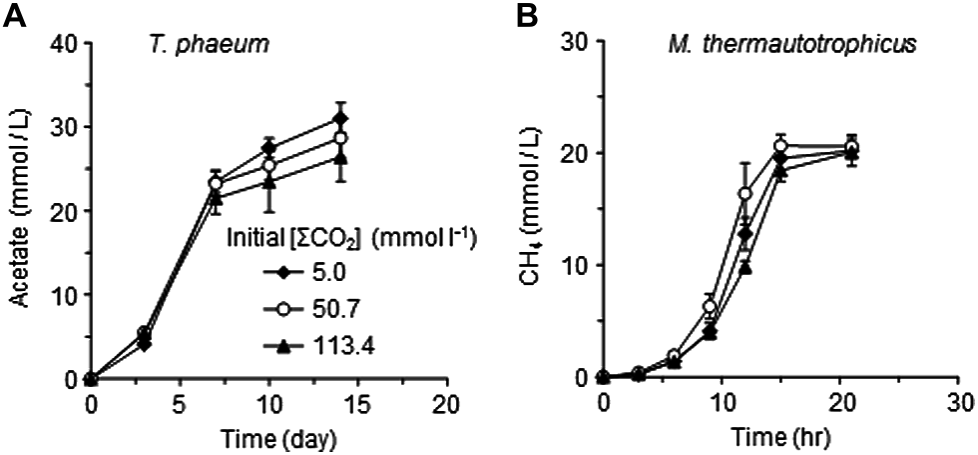
**Effects of CO_**2**_ concentrations on the pure cultures of *T. phaeum***(A)** and *Methanothermobacter thermautotrophicus***(B)**.** Data are presented as the means of three independent cultures, and error bars represent SDs.

One possible explanation for the suppressive effects of CO_2_ on the syntrophic methanogenesis is the susceptibility of *Methanothermobacter thermautotrophicus* and/or *T. phaeum* to some environmental alterations induced by increased CO_2_ or to CO_2_ itself. To evaluate this possibility, pure cultures of *Methanothermobacter thermautotrophicus* and *T. phaeum* were cultivated in media with different [ΣCO_2_]_initial_ (**Figure 5**). No significant differences were observed for the growth of both *Methanothermobacter thermautotrophicus* and *T. phaeum* under the different CO_2_ conditions tested. These results suggest that elevated CO_2_ concentrations negatively affect the microbial activity only when *Methanothermobacter thermautotrophicus* and *T. phaeum* are in a syntrophic relationship.

### EFFECTS OF CO_2_ CONCENTRATIONS ON THE THERMODYNAMICS OF EACH REACTION

The other possible explanation for the suppression of syntrophic methanogenesis by elevated CO_2_ concentration is alterations of thermodynamic conditions of each microbial reaction. A minimum energy required for biochemical energy conversion is estimated at around –20 kJ mol^-1^ (Schink, 1997), while some anaerobic microorganisms have been reported to thrive under more thermodynamically restricted conditions (Jackson and McInerney, 2002; Nauhaus et al., 2002). The value was estimated from the energetics of ATP formation (around -70 kJ mol^-1^ under the physiological conditions; Jetten et al., 1991; Tran and Unden, 1998) and the number of protons transported to ATP formation (between 3 and 4; Maloney, 1983; Stock et al., 1999). Since syntrophic methanogenesis from acetate is one of the least exergonic microbial metabolisms (Schink, 1997), it is no wonder that only slight perturbations on the thermodynamics induce deteriorations of the syntrophic methanogenesis.

**FIGURE 6:**
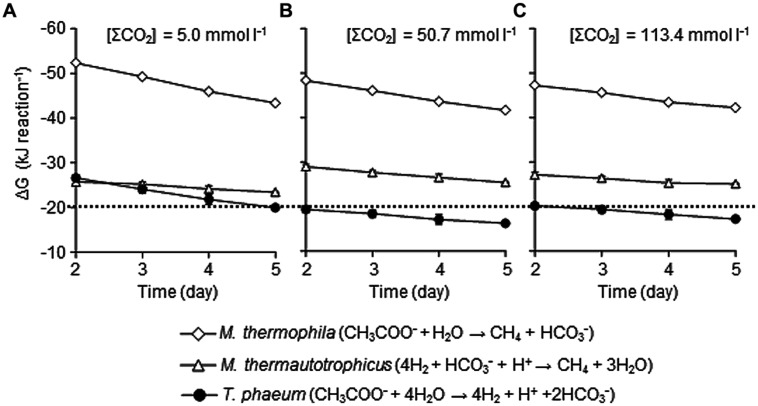
**Effects of CO_**2**_ concentrations on the Gibbs free energy change (ΔG) of metabolisms of each microorganism under the conditions with the [ΣCO_**2**_]_****initial****_ of 5.0 **(A)**, 50.7 **(B),** or 113.4 mmol l^**–1**^**(C)**.** The ΔG values were calculated using metabolite concentration data presented in **Figure 1**. The dotted line represents ΔG value of –20 kJ/reaction. Data are presented as the means of three independent cultures and error bars represent SDs.

To evaluate the influences of elevated CO_2_ concentrations on the thermodynamic properties, ΔG values of metabolic reactions conducted by each microorganism in the model consortium were determined using the data-set of metabolite concentrations shown in **Figure 1**. The ΔG values of the aceticlastic methanogenesis conducted by *Methanosaeta thermophila* were not significantly influenced by the elevated CO_2_ concentrations (**Figure 6**). The average ΔG values during the logarithmic growth phase (day 2–5) with the [ΣCO_2_]_initial_ of 5.0, 50.7 and 113.4 mmol l^-1^ were -47.7 ± 3.5, -44.9 ± 2.6, and -44.6 ± 2.0 kJ mol^-1^, respectively, which are substantially lower than the ΔG value required for microbial energy acquisition.

The ΔG values of the hydrogenotrophic methanogenesis catalyzed by *Methanothermobacter thermautotrophicus* were also largely not altered with different CO_2_ settings and were constantly lower than -20 kJ mol^-1^ (**Figure 6**). The average ΔG values during the logarithmic growth phases with the [ΣCO_2_]_initial_ of 5.0, 50.7, and 113.4 mmol l^-1^ were -24.6 ± 1.0, -27.2 ± 1.0, and -26.0 ± 1.2 kJ mol^-1^, respectively. Since CO_2_ is the substrate for hydrogenotrophic methanogenesis, lower ΔG values under the higher CO_2_ conditions are expected. However, the decrease in H_2_ partial pressures under the higher CO_2_ conditions (**Figure 1C**) compensates for the positive effects of increase in CO_2_ concentration.

On the contrary, elevation of CO_2_ concentrations significantly influenced the ΔG values of the acetate oxidation reaction performed by *T. phaeum* (**Figure 6**). While the average ΔG value during the logarithmic growth phases with the [ΣCO_2_]_initial_ of 5.0 mmol l^-1^ (-23.1 ± 2.7 kJ mol^-1^) was less than the borderline ΔG value of -20 kJ mol^-1^, those with the [ΣCO_2_]_initial_ of 50.7 and 113.4 mmol l^-1^ (-17.8 ± 1.3 and -18.7 ± 1.4 kJ mol^-1^, respectively) exceeded the borderline. As acetate oxidation reaction produces 2 mol of CO_2_ from 1 mol of acetate, it is rational that this reaction is strongly influenced by the elevation of CO_2_ concentration. The decrease in the partial pressure of H_2_, the other metabolic product of acetate oxidation, is expected to compensate for the negative effects of increase in CO_2_. However, the decrease in H_2_ partial pressure would be limited by the minimum threshold for H_2_ consumption by *Methanothermobacter thermautotrophicus*. The minimum thresholds for H_2_ utilization by hydrogenotrophic methanogens have been reported as around 5–10 Pa (Lovley, 1985; Thauer et al., 2008). However, considering the energy required for active growth, H_2_ partial pressure of around 10–15 Pa observed in the increased CO_2_ conditions in this study may be the minimum H_2_ threshold for the syntrophic interaction. Actually, if the H_2_ partial pressure in the cultures with [ΣCO_2_]_initial_ of 113.4 mmol l^-1^ at the logarithmic growth phase (day 5) becomes 10 Pa, the ΔG value becomes > -20 kJ mol^-1^ (-19.7 ± 0.3 kJ mol^-1^) from the actual value of -25.1 ± 1.4 kJ mol^-1^ (with H_2_ partial pressure of 16.4 ± 1.7 Pa). These results clearly demonstrated that high concentrations of CO_2_ thermodynamically constrain the acetate oxidizing reaction, which results in the deterioration of syntrophic methanogenesis from acetate.

## CONCLUSION

This is the first paper to evaluate the influence of elevated CO_2_ concentration on the two different methanogenic acetate degradation pathways, namely aceticlastic and syntrophic pathways, using a model microbial consortium. As expected from the observations based on *in situ* environments with complex microbial communities, high concentrations of CO_2_ suppressed the syntrophic pathway rather than the aceticlastic pathway. Thermodynamic calculations revealed that the acetate oxidation reaction is more intensely constrained by elevated CO_2_ concentrations. This study exemplified the importance of even slight changes in the ΔG values of microbial metabolisms in anaerobic biota. Furthermore, this study demonstrated that the construction of model microbial consortia is useful for assessing competitive interspecies interactions even in anaerobic, methanogenic environments.

## AUTHOR CONTRIBUTIONS

Souichiro Kato, Tomoyuki Sato, and Yoichi Kamagata designed the research. Souichiro Kato, Rina Yoshida, Takashi Yamaguchi, Tomoyuki Sato, and Isao Yumoto carried out the experiments and analyzed the data. Souichiro Kato and Yoichi Kamagata wrote the manuscript.

## Conflict of Interest Statement

The authors declare that the research was conducted in the absence of any commercial or financial relationships that could be construed as a potential conflict of interest.
